# Effect of Different Soft Segment Contents on the Energy Storage Capacity and Photo–Thermal Performance of Polyurethane-Based/Graphene Oxide Composite Solid–Solid Phase Change Materials

**DOI:** 10.3390/polym14235161

**Published:** 2022-11-27

**Authors:** Jiawei Wang, Zihua Wu, Huaqing Xie, Tingting Wang, Yuanyuan Wang, Yueming Huang, Lan Dong

**Affiliations:** 1 School of Energy and Materials, Shanghai Polytechnic University, Shanghai 201209, China; 2 Shanghai Engineering Research Center of Advanced Thermal Functional Materials, Shanghai 201209, China

**Keywords:** photo–thermal conversion polyurethane, different soft segment content, energy storage capacity, shape-stabilized

## Abstract

A series of polyurethane/graphene oxide (PU/GO) solid–solid phase change materials (SSPCMs) were synthesized by using GO as a light-absorbing filler and polyethylene glycol (PEG) as a phase change matrix. The effects of PEG content on the energy storage capacity, thermal stability and photo–thermal conversion performance of PU were investigated. The results show that the form-stability of PU/GO decreases while the phase change enthalpy and photo–thermal conversion efficiency of PU/GO increases with the increasing PEG content. The introduction of a very low content of GO can maintain comparable energy storage density and greatly improve light absorption by reasonably modulating the soft segment contents. The PU/GO composite with 92 wt% PEG has a phase change enthalpy of 138.12 J/g and a high photo–thermal conversion efficiency (87.6%). The composite solid–solid PCMs have great potential for effective energy storage and solar energy utilization.

## 1. Introduction

As a kind of clean and abundant renewable energy, solar energy has received a great deal of attention, and how to efficiently store and utilize solar energy have always been hotspots of researchers [1,2]. Phase change materials (PCMs) are functional materials which can efficiently store the thermal energy converted from solar energy [3]. Moreover, the high-efficiency heat collection and storage of solar energy using PCMs is simple to operate and has great economic feasibility [4,5]. Properties such as energy storage capacity, photo–thermal conversion and shape-stability have become the focus of research on PCMs. Polyethylene glycol (PEG) has the advantages of high energy storage density, wide phase transition temperature range, low or negligible subcooling, and is non-toxic and non-corrosive [6,7,8]. Therefore, PEG has become one of the most promising solid–liquid organic PCMs. However, thermal conductivity, thermal stability and light absorption of PEG are very poor, which have restricted the practical application of PEG [7]. In order to solve the above-mentioned inherent drawbacks of PEG, various light-absorbing fillers were introduced to prepare PEG-based composite PCMs.

Among various supporting fillers, graphene oxide (GO) is a nano-structured material with high thermal properties, superior electrical conductivity and mechanical properties [9]. The addition of GO can improve the light absorption ability of PCMs [10] and prevent its liquid phase leakage [11]. Qi et al. [12] prepared PEG/GO composite PCMs with GO as supporting material by a simple mixing and impregnation process. The shape of PEG/GO composite PCMs is mainly stabilized during the solid–liquid phase transition. The PEG/GO composite has a maximum weight percent of 96% PEG and no leakage occurs at temperatures up to 150 °C, which is much higher than the melting temperature of PEG. Ana et al. [13] used poly (propylene glycol fumarate) (PPF) to achieve non-covalent functionalization of PEG-GO composites. The presence of strong hydrogen bonding interactions between the ester group of the PPF and the OH moieties of PEG-GO significantly increased the thermal stability of the matrix. He et al. [14] obtained polyethylene glycol/graphene nano-platelet (PEG/GNPs) composites by a temperature-assisted solution blending method. The results showed that the thermal conductivity of PEG composite PCMs increased by 146% and the phase change enthalpy decreased by only 6.3% with the addition of 2 wt% GNPs. On the basis of this study, Yang et al. [15] also introduced a mixed filler composed of BN and a very small amount of GNP into PEG through a simple solution mixing process to make a PEG/GNP/BN composite PCMs. The thermal conductivity can be synergistically enhanced by the introduction of 1 wt% GNP which helps to form an improved and refined BN thermal pathway. The thermal conductivity of the final samples PEG/GNP/BN composite PCMs with 30 wt% BN and 1 wt% GNP reached 1.33 Wm^−1^ K^−1^, which were 336% higher than those of pure PEG, respectively. Li et al. [16] chose GO as filler to prepare GO/PEG composite PCMs by ultrasonic-assisted physical blending. The results showed GO/PEG composite PCMs exhibit better photo–thermal conversion performance than that of pure PEG. When the content of GO filler is 15 wt%, the photo–thermal conversion efficiency of GO/PEG composite PCMs is as high as 87.3%. Jiang et al. [17] prepared graphene/polyethylene glycol composites (G-PEGs) by the solution blending method. The results showed the phase change enthalpy and the thermoelectric output steady-state current time of G-PEGs gradually increased with the increase of PEG content, which indicates that the heat storage capacity of the composite PCMs is influenced by the soft segment.

Polyurethane (PU) is obtained by connecting the soft and hard segments beginning-to-end via a condensation reaction with polyethylene glycol as the soft segment and isocyanate as the hard segment. Isocyanates that can be combined with polyethylene glycol to synthesize polyurethane include 4,4′-methylene diisocyanate (MDI) [18], isophorone diisocyanate (IPDI) [19], cyclohexane diisocyanate (HMDI) and so on. The polyurethane-based phase change materials (PUPCMs) obtained by this reaction have high phase change enthalpy and wide phase transition temperature range. At the same time, since the PEG segments are connected to the hard segments and form a network structure, the shape of the PU is stable during the phase transition. Lu et al. [20] used PEG as a functional group, hexamethylene diisocyanate trimer (HDIT) as a cross-linking agent and a supporting framework to obtain a polyurethane-based solid–solid phase change material (PUSSPCM) through a one-step solvent-free synthesis method. Only 5 wt% content of HDIT can provide strong support for the acquisition of PEG segments in the phase transition temperature range, and the obtained PCMs have no leakage at 80 °C (above the melting temperature of PEG). It can be seen from above that polyurethane solid–solid PCMs have good shape and thermal stability and high heat storage, but the low efficiency of heat transfer performance and photo–thermal conversion limits the application of the PU. Pielichowska et al. [21] obtained graphene-modified polyurethane-based phase change materials (GPUPCMs) by an in situ polymerization method. Compared to GPUPCMs with chain extender, polyurethane without chain extender exhibited higher thermal stability. Moreover, the phase change enthalpy of graphene-modified PUPEG is 140.8 J/g, which is 7.7 J/g higher than that of unmodified PUPCMs. Zhou et al. [22] used PEG as a soft segment and hexamethylene diisocyanate (HDIB) as a cross-linking agent to synthesize cross-linked reduced graphene oxide/polyurethane solid–solid phase change materials (RGO/PUPCM) by a facile solvothermal treatment. Different from the direct physical mixing method, the reduction with HDIB and GO achieved physical and chemical cross-linking to give the samples good shape. The temperatures of all the samples rose with the increasing of RGO content in the time curves under light irradiation. The photo–thermal efficiency of PCMs is 78.7% with the 9 wt% RGO loading. However, the phase change enthalpy is only 107.2 J/g. This is mainly because too many hard segments reduce the crystallinity of PEG as a soft segment in PU, and the hard segments dispersed in the soft domains act as impurities and interfere with the crystal growth of PEG [23,24]. Since graphene and organic polymers are incompatible, it is difficult to form homogeneous composite PCMs by adding too much graphene, so it eventually leads to a decrease in thermal storage capacity. Li et al. [25] synthesized cellulose-graft-PEG copolymers, and the obtained graft copolymers exhibited solid–solid phase transition behavior with high thermal storage density and good thermal stability. It was found that the phase transition enthalpy could be adjusted from 80.3 J/g to 171.1 J/g by changing the content of PEG side chains. Therefore, the phase change enthalpy of PCMs can be improved by modulating the soft segment content, which will directly enhance the energy storage capacity of PCMs. Moreover, the energy storage capacity will affect the photo–thermal conversion performance of PCMs, so changing soft segment content will have a certain effect on the photo–thermal conversion capability.

In this work, PU/GO composite PCMs were prepared by in situ polymerization. The effects of different soft segment contents on the performances of PU/GO composite PCMs such as thermal conductivity, form-stability, energy storage capacity and light absorption were studied. The introduction of 1 wt% GO in PU/GO composite PCMs can help to realize strong light absorption ability and the photo–thermal conversion efficiency (87.6%). The preparation of solid–solid PCMs with high storage density (138.12 J/g) and good light absorption can propose a simple and new means of photo–thermal collection and solar energy storage.

## 2. Experimental Section

### 2.1. Materials

Natural Flake graphites (NFG, Particle), potassium permanganate (KMnO_4_) and polyethylene glycol (PEG, M_n_ = 10,000) were all purchased from Shanghai National Medicines Co. Inc., concentrated sulfuric acid (H_2_SO_4_), hydrochloric acid (HCl), concentrated nitric acid (HNO_3_), hydrogen peroxide (H_2_O_2_), N, N-Dimethylformamide (DMF) and 4,4′-methylene diisocyanate (MDI) were all obtained from Shanghai Aladdin Bio-Chem Technology Co., Ltd., and 1,4-butanediol (BDO) was supplied by Shanghai Medical Chemical Reagent Co., Inc. (Shanghai, China). 

### 2.2. Preparation of GO

GO was prepared from natural flake graphite according to a modified Hummers’ method [26]. Firstly, concentrated HNO_3_ and H_2_SO_4_ in a volume ratio of 2:1 were mixed to form a mixed acid. The natural flake graphite (3.0 g) was added into 25 mL mixed acid during the stirring process and reduced the temperature down to 24 °C. Then 4.0 g KMnO_4_ was added into the solution which maintained the temperature at 30 °C for 1 h. The mixture was heated to 65 °C and kept for 18 h. Then 100 mL of distilled water containing 10% H_2_O_2_ was added to the previous mixture. Stirring process continued until the solution became bright yellow. The oxidized graphite was obtained after centrifugation, neutralization, washing and drying.

### 2.3. Synthesis of PU/GO Composites

**Figure 1:**
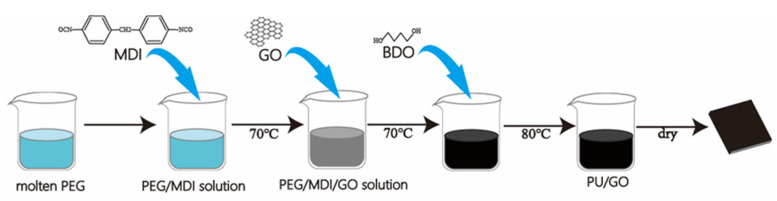
The synthetic route of PU/GO.

The in situ polymerization of PU/GO composites was carried out in two steps (Figure 1): in the first step, a predetermined amount of PEG and MDI were mixed in the DMF which was stirred in a nitrogen atmosphere at 70–75 °C. Then GO was added into water for ultrasonic dispersion, and 10 mg/mL GO aqueous dispersion was prepared. A certain amount of prepared GO water dispersion droplets was added to the NCO terminated modified PEG for further reaction for 2 h. In the next step, a certain quality of BDO was added into the PEG/MDI/GO solution and the reaction was continued at 80 °C for 2 h for the chain extension. Then, the reaction mixture was cast on the prepared mold and further vacuum dried at 80 °C for two days to ensure the complete volatilization of DMF. Before the test, the samples were stored in vacuum at room temperature for two weeks. The prepared PU/GO samples with 1 wt% GO are named according to the PEG contents of 80 wt%, 85 wt%, 89 wt%, 92 wt% and 95 wt%, as shown in Table 1.

### 2.4. Characterization

The micromorphology and chemical structure of the samples were analyzed by a Bruker D8-Advance X-ray diffractometer (XRD) and a Hitachi S-4800 scanning electron microscope (SEM) under an acceleration voltage of 10 kV. The infrared spectra of powders were recorded on a Brugg Tensor27 Fourier transform infrared (FT-IR) instrument, all spectra in the range of 500–4000 cm^−1^. The heat capacity of the samples was measured on a PerkinElmer differential scanning calorimeter (DSC). The temperature rise of the samples was investigated by an Agilent-34970A temperature recorder with J-type thermocouple. The light absorption performance of samples was carried out on an ultraviolet—visible(UV—vis) spectrometer. Zolix xenon arc lamps were used as simulated sunlight.

## 3. Results and Discussion

### 3.1. The Chemical and Structural Analysis of PCMs

**Figure 2:**
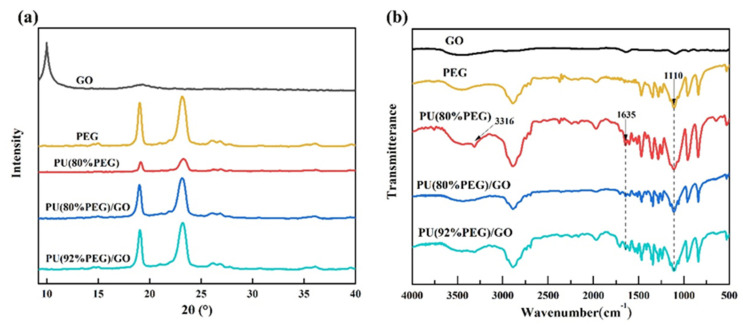
(**a**) XRD patterns of PEG, PU and PU/GO; (**b**) FT-IR spectra of PEG, PU and PU/GO.

Figure 2a shows the XRD patterns of PU and PU/GO composites. The high diffraction peaks at 2θ = 19.1° and 23.2° indicate the good crystallinity of PEG. After the reaction of PEG and MDI, the deceased intensity of the diffraction peak of PU(PEG80%) indicates that the crystallinity becomes lower. When GO was added into PU(PEG80%), the intensity of the diffraction peak increased obviously, which indicates that the crystallinity is improved. This is mainly because some functional groups of GO interact with MDI which reduced the crosslinking degrees of PU. In addition, the crystallization of PEG may be influenced by the crosslinking degrees of PU [27]. PU/GO exhibit all the characteristic peaks of PEG, but no new diffraction peaks appear. The results indicate the addition of GO is the only physical modification, and no new substance is produced. The structure of GO, PEG, PU and PU/GO composites confirmed by the FT-IR method are shown in Figure 2b and Table 2. 

In the GO spectrum, the peaks at 1638 cm^−1^ and 1092 cm^−1^ correspond to their C=O and C-O tensile vibration. The peaks at 1061 cm^−1^, 1099 cm^−1^ and 1148 cm^−1^ in the pure PEG spectrum correspond to C-O tensile vibration. After the reaction of MDI with PEG, a new absorption peak emerges in PU at 1635 cm^−1^, indicating that the synthesis of polyurethane is successful. The absorption peak of the amino group of the PU without GO obviously appears at 3316 cm^−1^. The absorption peak of PU gradually disappears after the addition of GO, which was mainly due to the reaction of -NCO in MDI with -COOH or -OH of GO [28]. The strong peaks at 1110 cm^−1^ in PU and PU/GO spectra are attributed to the C-O tensile vibration of the soft segment PEG. After being mixed with the GO, the spectrums of PU/GO show the characteristic peaks of PU with no new absorption peaks. This is mainly because the content of GO is too small. As can also be seen from Table 2, there is no significant change in the position of the vibrational bands in the samples modified with GO, indicating that no other new functional groups are generated.

### 3.2. Micromorphology Analysis of PCMs

**Figure 3:**
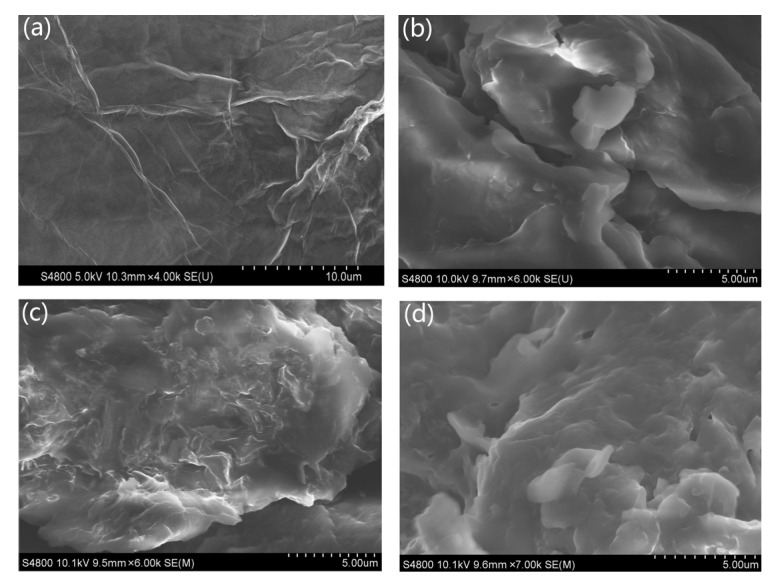
SEM images of the surface: (**a**) GO; (**b**) PU(80%PEG); (**c**) PU(80%PEG)/GO; (**d**) PU(92%PEG)/GO.

Figure 3 shows the SEM images of GO, PU and PU/GO to explore the effect of soft content on the microstructure of composite PCMs. Figure 3a shows GO has multilayer structures. It can be seen from Figure 3b that the surface of PU is relatively smooth with obvious edges. Figure 3c,d manifest the SEM images of PU/GO, which shows that the PU surface will present irregular protrusions when the soft content is lower than 80 wt%. These protrusions may contribute to enhancing the thermal stability of the composite PCMs. The microstructure of PU/GO will turn into laminated structure when the soft content is 92 wt%. The surface of PU/GO composites becomes smoother with the increasing of PEG content. This is because of the decrease of MDI content, which leads to the reduction of the reaction between -NCO in MDI and GO [29]. 

### 3.3. Thermophysical Properties of PCMs

#### 3.3.1. Thermophysical Properties of PU/GO

**Figure 4:**
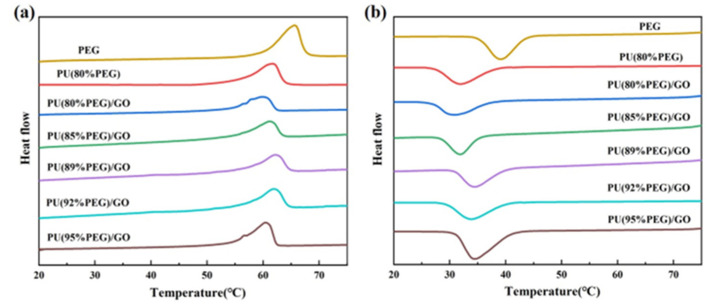
DSC curves of PU/GO during heating (**a**) and cooling (**b**).

The phase change temperatures and phase change enthalpies of PEG, PU and PU/GO composites are represented in DSC curves and data (Figure 4 and Table 3). Due to the hydrogen bond interaction between GO and PEG molecules, the melting point (Tm) and crystallization point (Tc) of the PU/GO composites slightly decrease after adding GO into PU [30]. However, with the increase of PEG content, the Tm of the composites remains almost constant. The phase change enthalpies and the degree of crystallinity (Xc) of the composite PCMs increase with the rise of PEG content because the increase of soft segment contents promotes a neater arrangement of molecular chains and better crystallinity. When the PEG content is 95 wt%, the phase change enthalpy of the PU/GO composites is high up to 157.43 J/g, which is equal to 90% of PEG. These results indicate PU/GO composites have a strong thermal storage capacity and can satisfy practical requirements.

#### 3.3.2. Thermal Stability of PU/GO

**Figure 5:**
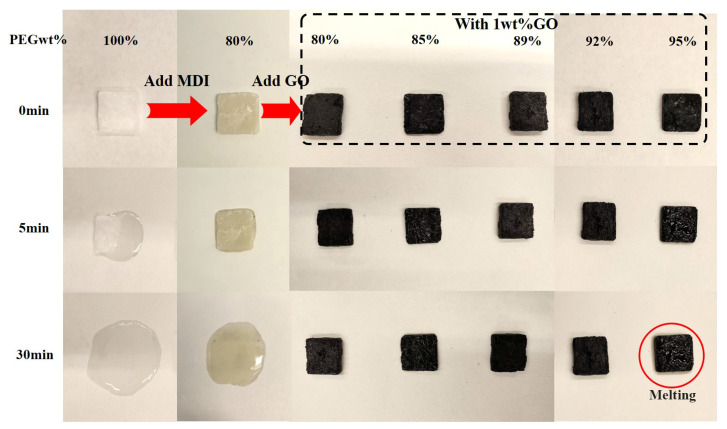
Digital images of PEG, PU and PU/GO at 80 °C for 30 min.

Figure 5 shows digital images of samples heated on a heating plate at different times. Pure PEG is a white one at room temperature. As the heating time increases, PEG flows while PU remains in its form at 5 min and completely melts into liquid at 30 min. This is mainly because when the system is heated, the crystallized PEG will melt into irregular chain segments which tend to flow in all directions. MDI can be chemically bonded to PEG to form PU polymers so that the movement of PEG is restricted. Therefore, the good compatibility of soft and hard segments can facilitate the absorption of PCMs and further avoid leakage. Figure 5 also shows that the surface color of PU/GO composites turns black after the addition of 1 wt% GO. Unlike PEG and PU, there is no leakage throughout the PU/GO composites during the heating time. This is mainly due to the grafting effect of GO and MDI. In addition, PU/GO with 92 wt% PEG holds its shape well while PU/GO with 95 wt% PEG melts slightly. This indicates that the soft segment content has an influence on the shape stability of PU/GO composite PCMs. 

**Figure 6:**
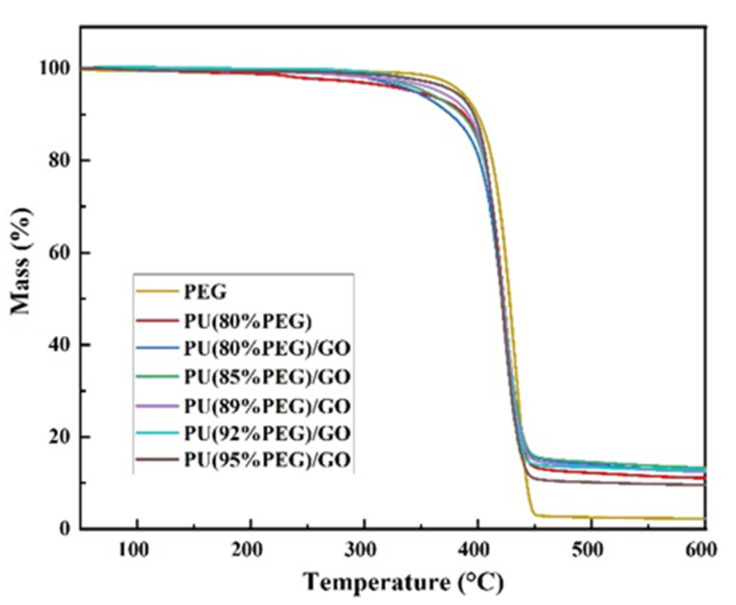
TG curves of PEG, PU and PU/GO.

Figure 6 and Table 3 reveal the thermal stability of PEG, PU and PU/GO composites. There is no significant weight loss when the temperature is between 60 °C and 250 °C. This phenomenon indicates that the PCMs are stable at common operating temperature. Whereas when the temperature is between 250 °C and 350 °C, the PCMs exhibit slight decomposition related to the degradation of urethane groups on polyurethane. When 1 wt% GO is added, the maximum weight loss decreases to 83.8%, indicating that the addition of GO will enhance the thermal stability of PU. As can be seen from Table 4, the temperature increases at 5% mass loss and 10% mass loss with the increase of PEG content, and the maximum mass loss also increases with the increase of PEG content. This is mainly due to the thermal decomposition of the PEG segment.

#### 3.3.3. Thermal Transfer Property of PU/GO

**Figure 7:**
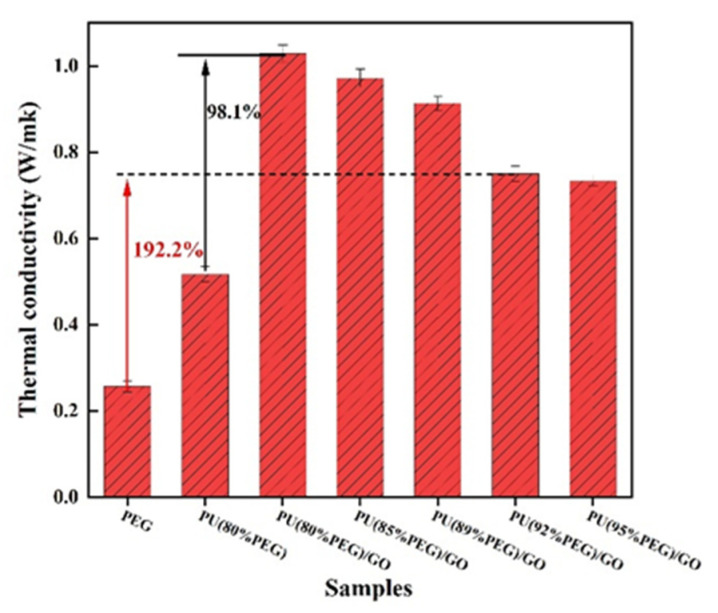
Thermal conductivities of PU and PU/GO.

Figure 7 shows the thermal conductivities of PU and the composite PCMs. The thermal conductivity of PU(PEG80%)/GO is 1.03 Wm^−1^ K^−1^, which shows relative enhancement of 98.1% compared with PU(PEG80%). This is because that the isocyanate in MDI is covalently grafted with GO to greatly improve the thermal conductivity of PU. The thermal conductivity of the composite PCMs gradually decreased as the PEG content increased. This is mainly because the low thermal conductivity of PEG (0.251 Wm^−1^ K^−1^) and the addition of much PEG leads to an increase in the voids of the composite material, which increases the interfacial thermal resistance and leads to a decrease in thermal conductivity. When PEG content is 92 wt%, the thermal conductivity of PU/GO can also reach as high as 0.751 Wm^−1^ K^−1^, which is also 192.2% higher than that of PEG.

### 3.4. Photo–Thermal Energy Conversion

**Figure 8:**
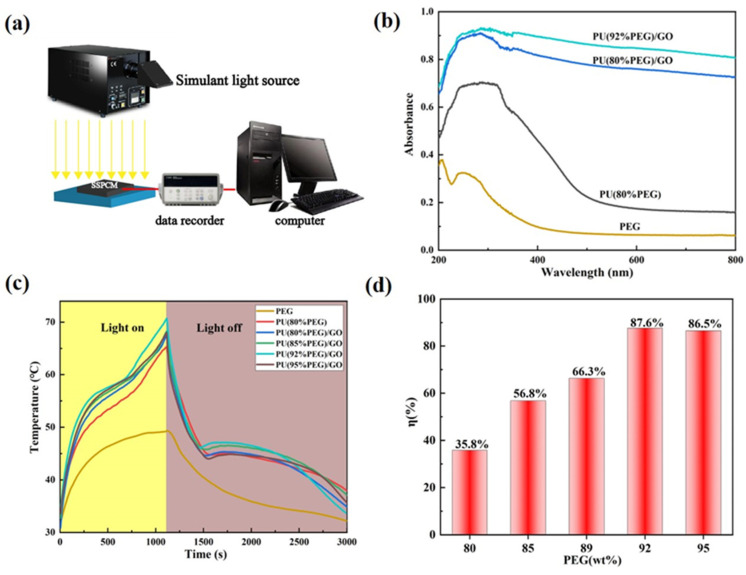
(**a**) Experimental diagram of photo–thermal energy conversion and storage test system; (**b**) absorption spectrum of PEG, PU and PU/GO; (**c**) temperature–time curves of PU/GO under simulated solar radiation at 100 mW/cm^2^; (**d**) photo–thermal conversion efficiency for PU/GO doped with different contents of PEG.

The diagram of experimental device in Figure 8a is designed to investigate photo–thermal energy conversion performance with xenon arc lamps as the solar simulator. The spectrophotometer is used to calibrate the intensity at 100 mW/cm^2^ for photo–thermal energy conversion and storage tests. Figure 8b concerns the UV-VIS-NIR spectra, it shows that PU/GO has stronger light absorption in the whole range, while pure PEG has no absorption peak. Therefore, as a light-absorbing filler, GO has a prominent effect on PU polymers. Under simulated solar radiation, photo–thermal energy conversion is mainly studied (Figure 8c). When the melting temperature of the composites was reached, an inflection point appeared in the temperature–time curve. The change is in line with the phase change temperature of the composites. When PEG content is less than 95 wt% in the PU/GO composites, the heating rate increases with PEG content increases, and the temperature of PU(PEG92%)/GO rises from 30 °C to 70 °C in 1100 s, which is 3 °C higher than PU(PEG95%)/GO at the same time. This is because the photo–thermal conversion performance is influenced by the thermal conductivity and the phase change enthalpy. When the heat transfer does dominate, increasing the soft segment content of PU/GO will limit the phonon transport and increase the interfacial thermal resistance, thus reducing the heating rate of PU/GO [31]. As can be demonstrated by the UV-vis-NIR curves, the absorbance of PU(PEG95%)/GO is slightly lower than PU(PEG92%)/GO (Figure 8b). Unlike PU and PU/GO composite PCMs, PEG has been maintained at 49 °C and there was no inflection point on the photo–thermal conversion curve, which indicates that PEG cannot reach its melting point and that the phase-transition process did not occur. After the removal of the solar simulator, the temperature of PU/GO dropped and another inflection point appeared. This indicates that the composite starts to release previously stored thermal energy and then transfers the energy to samples during the solidification process. The efficiency of photo–thermal energy storage can be calculated by the following equation:

(1) $$\eta =\frac{m\cdot \Delta H}{P\cdot A\cdot (T_{s}-T_{e})}$$

where *m* and Δ*H* are the weight and phase change enthalpy of the composites, *P* is the power of the simulated solar irradiation, *A* is the illuminated area of the composites, and *T_s_* and *T_e_* are the onset and termination points of the phase transition (Figure 8d). The photo–thermal conversion efficiency (*η*) was 87.6% when the PEG content was 92 wt%. This result confirms that PU/GO composites can efficiently be used for solar energy harvesting, photo–thermal conversion and thermal energy storage.

## 4. Conclusions

In this paper, PU/GO SSPCMs were prepared for the effective utilization of solar energy through photo–thermal conversion. PU/GO composite PCMs were fabricated by in situ polymerization of PU and GO. The results showed that the thermal conductivity of PU/GO with different PEG content increased from 42.3% to 98.1% after the addition of a small amount of GO. The energy storage capability and photo–thermal conversion efficiency of PCMs was improved. In addition, GO has a prominent effect on PU polymers in light absorption and photothermal conversion. The PU/GO composite doped with 92 wt% PEG shows the highest heating rate and photo–thermal conversion efficiency is 87.6%. The phase change enthalpy of PU/GO composites increased with the increase of soft segment content. When the PEG content is 95%, the PU/GO composites have a phase change enthalpy approach to 157.43 J/g. Our results can provide a new means of the solar energy storage and conversation.

## Figures and Tables

**Table 1:** The compositions of PU/GO.

Compositions	Samples	PEG (wt%)	GO (wt%)
PEG	PEG	100	0
PU	PU(80%PEG)	80	1
PU/GO	PU(80%PEG)/GO	80	1
	PU(85%PEG)/GO	85	1
	PU(89%PEG)/GO	89	1
	PU(92%PEG)/GO	92	1
	PU(95%PEG)/GO	95	1

**Table 2:** The detailed compositions of the FT-IR spectra.

	GO	PEG	PU(80%PEG)	PU(80%PEG)/GO	PU(92%PEG)/GO
Vibration bands	Wavenumber (cm^−1^)				
N-H stretching	-	-	3316	3309	3304
C=O stretching	1638	-	1635	1640	1647
C-O stretching	1092	1099	1106	1105	1102

**Table 3:** DSC data of PEG, PU and PU/GO.

Samples	GO (wt%)	PEG (wt%)	Tm (°C)	Tc	ΔHm	ΔHc	Xc (%)
PEG	0	100	65.13	39.33	174.6	169.2	100
PU(PEG80%)	0	80	61.84	32.77	103.62	97.98	74.2
PU(PEG80%)/GO	1	80	60.01	30.88	92.41	89.48	66.2
PU(PEG85%)/GO	1	85	61.26	31.82	111.7	103.7	75.3
PU(PEG89%)/GO	1	89	62.42	34.45	124.13	116.08	79.9
PU(PEG92%)/GO	1	92	61.81	33.67	138.12	129.14	86.0
PU(PEG95%)/GO	1	95	61.39	34.06	157.43	151.32	94.9

**Table 4:** TG data of PU and PU/GO in nitrogen.

Samples	Temperature at 5% Weight Loss (°C)	Temperature at 10% Weight Loss (°C)	Maximum Weight Loss (%)
PEG	385	400	97.2
PU(PEG80%)	343	387	86.2
PU(PEG80%)/GO	344	375	83.8
PU(PEG85%)/GO	353	385	84.1
PU(PEG89%)/GO	365	391	85.1
PU(PEG92%)/GO	379	395	85.7
PU(PEG95%)/GO	379	396	88.5
